# Supplementary figures and images for: The incidence of and risk factors for late presentation of childhood chronic kidney disease: A systematic review and meta-analysis
Source: PLoS One. 2020 Dec 31;15(12):e0244709. doi: 10.1371/journal.pone.0244709 (PMC7774987; doi:10.1371/journal.pone.0244709)

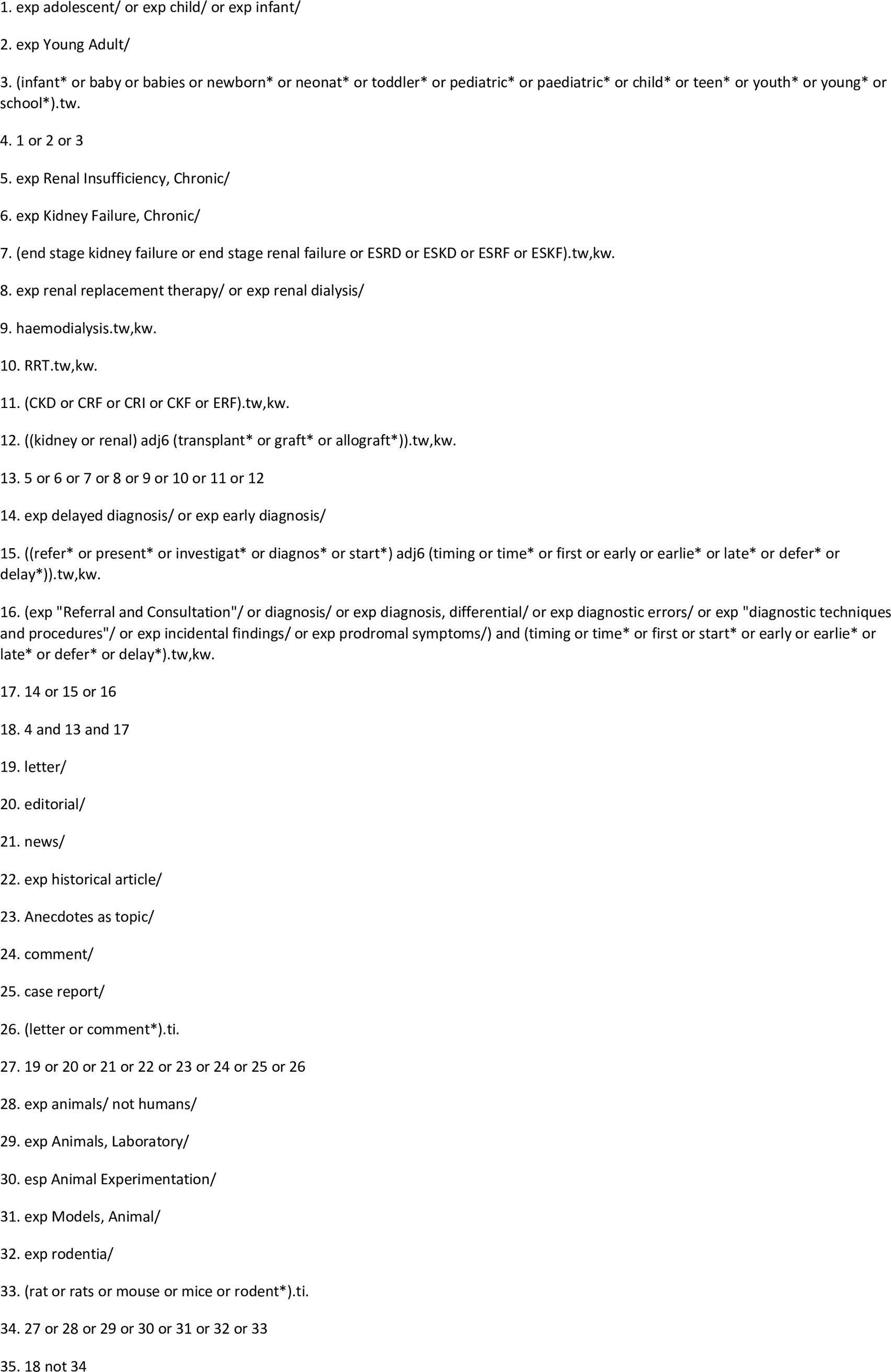

Supplement: S1 Fig — (TIF) [file pone.0244709.s002.tif]

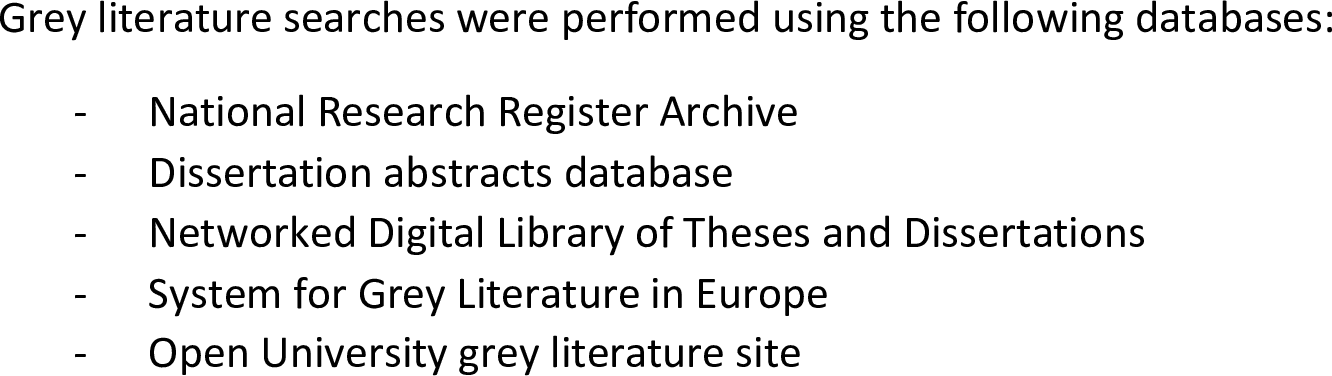

Supplement: S2 Fig — (TIF) [file pone.0244709.s003.tif]

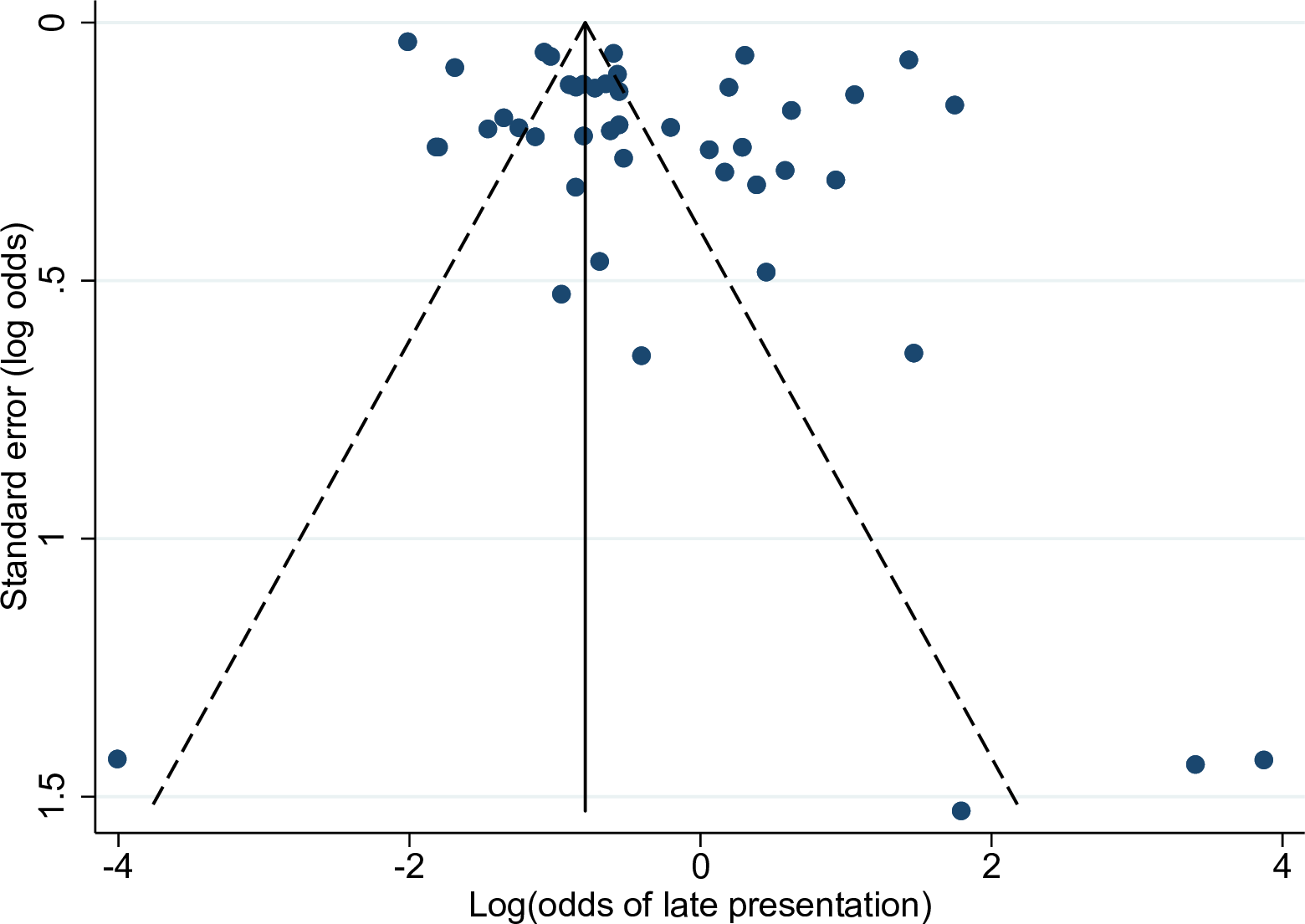

Supplement: S3 Fig — (TIF) [file pone.0244709.s004.tif]
