# Supplementary material for: The incidence of and risk factors for late presentation of childhood chronic kidney disease: A systematic review and meta-analysis
Source: PLoS One. 2020 Dec 31;15(12):e0244709. doi: 10.1371/journal.pone.0244709 (PMC7774987; doi:10.1371/journal.pone.0244709)
Supplement: S1 Table — (DOCX) [file pone.0244709.s005.docx]

SUPPLEMENTAL TABLE 1: Modified Newcastle-Ottawa Score

| **Criteria** | **Acceptable (star awarded)** | **Unacceptable (star not awarded)** |
| --- | --- | --- |
| **Representativeness of study cohort (external validity)** | Study truly or somewhat representative of children with exposure (chronic kidney disease or end-stage renal disease) from population studied i.e. all referrals with CKD/ESKD | Exclusion of sub-group of children (i.e. those who die or are transplanted)  Ward/specialist admissions only  Insufficient information to discern representativeness |
| **Source of patients** | All patients identified from same source (i.e. same hospital setting(s)) | No/unclear information concerning source of patients included  Patients drawn different sources |
| **Ascertainment of exposure** | CKD determined using recognised guideline (i.e. KDIGO/NKF-KDOQI) or clear description of study-specific definition used based on laboratory and/or clinical/radiological findings | No clear definition given  Self-report |
| **Comparability (analytic studies)- up to 2 stars awarded** | Results (relevant to study outcomes) adjusted for age and/or gender  Results (relevant to study outcomes) adjusted for additional confounding variable i.e. ethnicity | No confounders adjusted for in analysis |
| **Outcome of interest** | Clear description of end-stage kidney disease (ESKD) at presentation or late presentation/late referral (or synonyms thereof) and/or study-specfic definition given | Use of terminology (i.e. ESKD) but without clear/reference definition given  Self-report |

Abbreviations: CKD, Chronic Kidney Disease; ESKD, End-Stage Kidney Disease; KDIGO, Kidney Disease: Improving Global Outcomes; NKF-K/DOQI, National Kidney Federation- Kidney Disease Outcomes Quality Initiative.
