# Supplementary material for: The incidence of and risk factors for late presentation of childhood chronic kidney disease: A systematic review and meta-analysis
Source: PLoS One. 2020 Dec 31;15(12):e0244709. doi: 10.1371/journal.pone.0244709 (PMC7774987; doi:10.1371/journal.pone.0244709)
Supplement: S2 Table — Abbreviations: AKI, Acute Kidney Injury; ATN, acute tubular necrosis; CrCl, creatinine clearance; CRF, chronic renal failure; CKD, chronic kidney disease [Note: Terminology used reflects that of study. CRF and CKD terms used interchangeably]; ESRF, End-Stage Renal Failure; ESRD, End-Stage Renal Disease [Note: Terminology used reflects that of study. ESRF and ESRD terms used interchangeably and are synonymous with ‘kidney failure’]; eGFR, estimated Glomerular Filtration Rate; NAPRTCS, North American Pediatric Trials and Collaborative Studies; NR, not recorded; PN, Paediatric Nephrology; RRT, Renal Replacement Therapy [Note terminology used reflects that of study and is used interchangeably with Kidney Replacement Therapy]; SD, standard deviation; SES, socio-economic status; USRDS, United States Renal Data System. *Numbers of excluded patients are provided where available. (DOCX) [file pone.0244709.s006.docx]

SUPPLEMENTAL TABLE 2**:** Description of studies included in systematic review

| **Title** | **First author** | **Year** | **Country** | **Study design** | **Data collection** | **Study aim** | **Study length (years)** | **Population** | **Exclusions*** | **Study location** | **Age inclusion (years)** | **Eligibility for study inclusion** | **Sample size** |
| --- | --- | --- | --- | --- | --- | --- | --- | --- | --- | --- | --- | --- | --- |
| Chronic renal failure in children in Saudi Arabia | Mattoo^25^ | 1990 | Saudi Arabia | Retrospective single-centre | NR | To identify the common causes of CRF, assess the problems related to its treatment and discuss the therapeutic modalities available locally | 3.0 | Children referred to renal unit | NR | PN services (all) | <14 | ‘Irreversible’ creatinine >2mg/dL (180µmol/L) | 100 |
| Chronic renal failure in children in Asir region of Saudi Arabia | Al Harbi^26^ | 1997 | Saudi Arabia | Retrospective single-centre | Review of medical records | To determine incidence and causes of CRF in children in the region | 5.0 | Children admitted to hospital with chronic renal failure in time period | NR | Hospital admissions | <13 | Creatinine > 177µmol/L (2mg/dL) or >132µmol/L (1.5mg/dL) in children <2 years) >3 months | 62 |
| Etiology and outcome of chronic renal failure in Indian children | Gulati^27^ | 1999 | India | Prospective single-centre | Review of medical records | Analyse the aetiology, spectrum and outcome of chronic renal failure over a one year period | 1.0 | All new cases of paediatric chronic renal failure in time period | NR | PN services (all) | <17 | Creatinine >2mg/dL (180µmol/L) >3 months | 48 |
| Renal failure in children | Hafeez^28^ | 2002 | India | Prospective single-centre | Review of medical records | Assess the clinical pattern, aetiology and outcome of patients with CRF | 1.0 | Children attending nephrology department with chronic renal failure | NR | PN services (all) | <16 | eGFR <75ml/min/1.73m^2^ | 42 |
| Epidemiology of chronic renal failure in children: Data from the ItalKid project | Ardissino^29^ | 2003 | Italy | Prospective multi-centre | Registry data | To describe the ItalKid project and the basic epidemiological results of the first ten years | 10.0 | New registration of CKD from renal units | NR | PN services (all) | <20 | eGFR<75 ml/min/1.73m^2^; mean serum creatinine +3 SD if <1 year of age | 1197 |
| Chronic renal failure in Indian children | Hari^30^ | 2003 | India | Retrospective single-centre | Review of medical records | Examine the aetiology of chronic renal failure in children referred to a single-centre tertiary hospital | 7.0 | Patients diagnosed with chronic renal failure in time period | NR | PN services (all) | <19 | eGFR <50 ml/min/1.73m^2^ >3 months | 305 |
| Renal failure in Nigerian Children: factors limiting access to dialysis | Olowu^23^ | 2003 | Nigeria | Prospective single-centre | Review of medical records | Determine factors limiting ready access of Nigerian children with RF to dialysis | 5.0 | Children admitted to nephrology unit for renal failure | AKI | Hospital admissions | <16 | eGFR <60 ml/min/1.73m^2^ (or creatinine >120µmol/L) for 6 months | 21 |
| Chronic renal failure in children of Benin, Nigeria | Michael^31^ | 2004 | Nigeria | Prospective single-centre | Review of medical records | To report the pattern of childhood renal diseases seen at centre | 6.0 | All patients admitted to paediatric inpatient service with CKD | NR | Hospital admissions | <17 | eGFR <25 ml/min/1.73m^2^ | 24 |
| Analysis of 1268 patients with chronic renal failure in childhood: a report from 91 hospitals in China from 1990 to 2002 | Yang^32^ | 2004 | China | Retrospective multi-centre | Review of medical records | To investigate the epidemiology, natural history, clinicopatholical manifestations, treatment and outcome of hospitalised children with CRF | 13.0 | All children newly hospitalised for CRF with CrCl <50 | Missing records (n=39) | Hospital admissions | <15 | eGFR <50 ml/min/1.73m^2^ | 1229 |
| The major causes of chronic renal insufficiency in Syrian children: a one year, single-centre experience | Saeed^33^ | 2005 | Syria | Prospective, single-centre | Review of medical records | To determine the major causes, clinical expression, course and outcomes of CKD in Syrian children | 1.0 | All children presented to paediatric nephrology department with CKD | NR | PN services (all) | <16 | eGFR <50 ml/min/1.73m^2^ | 18 |
| Chronic renal failure in children in the Western area of Saudi Arabia | Kari^21^ | 2006 | Saudi Arabia | Retrospective, single-centre | Review of medical records | Provide epidemiological data about CRF in children in western province of Saudi Arabia | 3.8 | All children first seen at King Abdul Aziz University Hospital with CKD | NR | PN services (all) | <15 | eGFR <50 ml/min/1.73m^2^ | 66 |
| Chronic kidney diseases in Iraqi children | Hassan^22^ | 2007 | Iraq | Prospective single-centre | Review of medical records | To identify the aetiology of chronic disease in the studied group, to assess growth in relation to the degree of renal impairment, to study factors that influence progression of renal disease and highlight difficulties faced in management | 2.0 | All children referred to PN services | NR | PN services (all) | <18 | eGFR <50 ml/min/1.73m^2^ >3 months or less if clinical evidence of CKD | 110 |
| Etiology and outcome of chronic renal failure in hospitalised children in Ho Chi Minh City, Vietnam | Mong Hiep^35^ | 2008 | Vietnam | Retrospective, multi-centre | Review of medical records | To estimate the annual number of children diagnosed with CRF to determine the causes, describe associated symptoms at entry, analyse treatment modality and identify risk factors of mortality in hospitalised children with CRF. | 5.0 | Hospitalised children diagnosed with CKD in time period | NR | Hospital admissions | <19 | Clinical evidence of CKD for >3 months | 310 |
| NAPRTCS 2008 annual report | NAPRTCS^34^ | 2008 | USA | Prospective multi-centre | Registry data |  | 14.0 | Children newly registered with (voluntary) registry – data submitted from paediatric nephrology centres | Missing data | PN services (all) | <21 | eGFR <75 ml/min/1.73m^2^ | 6969 |
| Chronic kidney disease in children: the national paediatric hospital experience in Hanoi, Vietnam | Huong^37^ | 2009 | Vietnam | Retrospective, single-centre | Review of medical records | Estimate the number of annually hospitalised children diagnosed with CKD, present how the economic condition of the patient is an important limiting factor for acceptance of RRT, identify factors for treatment refusal, suggest simple ideas to improve prevention of CKD and health care of paediatric patients in Vietnam | 5.0 | Hospitalised children diagnosed with CKD (first admission) | NR | Hospital admissions | <18 | Clinical assessment of CKD and creatinine >150µmol/L (1.7mg/dL) for >3months | 152 |
| Chronic Renal Failure in Jamaican children- an update (2001-2006) | Miller^39^ | 2009 | Jamaica | Retrospective multi-centre | Review of medical records and questionnaire to local hospitals for non-referred cases of CRF. | Document the incidence, epidemiology and aetiology of CRF in Jamaican children < 12 years old | 6.0 | All incident cases paediatric of chronic renal failure | NR | Country-wide survey/PN services (all) | <12 | eGFR <50 ml/min/1.73m^2^ for 3 months or less if clinical evidence | 18 |
| Chronic Kidney Disease in Southwestern Iranian Children | Ahmadzadeh^38^ | 2009 | Iran | Retrospective single-centre | Review of medical records | Determine the aetiology of CKD in children referred to centre | 10.1 | Patients newly diagnosed with chronic renal failure | Follow up less than <3 months and incomplete data | PN services (all) | <17 | eGFR <60 ml/min/1.73m^2^ >3 months | 139 |
| Chronic kidney disease in children in Turkey | Bek^36^ | 2009 | Turkey | Retrospective multi-centre | Proforma collected for cases | To determine the annual incidence of CKD in children in 2005 | 1.0 | New cases of CKD | NR | PN services (all) | <19 | eGFR <75 ml/min/1.73m^2^ ≥3 months | 282 |
| Clinical characteristics and outcomes of children with stage 3-5 CKD | Mong Hiep^40^ | 2010 | Belgium | Prospective multi-centre | Registry data | Provide up to date reliable information on the clinical characteristics and nature of primary renal diseases, to analyse modalities of treatment and to evaluate the outcomes such as comorbidities, progression to ESRF and causes of death for incident children with CKD | 5.0 | All children newly diagnosed with CKD | NR | PN services (all) | <20 | eGFR <60 ml/min/1.73m^2^ | 143 |
| Epidemiology of chronic kidney disease in children in Serbia | Peco-Antic^41^ | 2011 | Serbia | Prospective multi-centre | Registry data | To determine the epidemiology of earlier stages of CKD in Serbian children | 10.0 | All children referred with GFR<90 for at least 3 months | NR | PN services (all) | <19 | eGFR<90 ml/min/1.73m^2^ (or creatinine >2 SD in <2years) for ≥3 months | 336 |
| Chronic kidney disease in children: a report from a tertiary care centre over 11 years | Gheissari^42^ | 2012 | Iran | Retrospective single-centre | Review of medical records | To evaluate the incidence and aetiology of CRF in children in a tertiary care centre in Isfahan | 10.1 | Children hospitalised with new CKD | NR | Hospital admissions | <19 | eGFR <60 ml/min/1.73m^2^ for >3 months | 268 |
| Chronic Kidney disease during a twelve-year period at a Tertiary Health Institution | Paripović^43^ | 2012 | Serbia | Retrospective single-centre | Review of medical records | To analyse the aetiology, stages and associated complications of CKD at the time of referral for patients treated in a tertiary healthcare setting. | 12.0 | Children newly referred to tertiary hospital with CKD | NR | PN services (all) | <19 | eGFR <90 ml/min/1.73m^2^ | 97 |
| The demographic characteristics of children with CKD stages 3-5 in SE England over a 5 year period | Kim^46^ | 2013 | UK | Retrospective single-centre | Review of medical records | To report the demographic characteristics of moderate-severe CKD at a single-centre | 5.0 | Incident children with CKD 3-5 referred to tertiary hospital | Children with functioning kidney transplants | PN services (all) | <16 | eGFR <60 ml/min/1.73m^2^ for 12 months | 141 |
| Pattern of renal diseases in children presented to King Abdulaziz University Hospital | Alsaggaf^45^ | 2013 | Saudi Arabia | Retrospective single-centre | Review of medical records | To evaluate the pattern of renal disease and outcomes in children presenting to hospital | 8.0 | All renal cases presenting to paediatric nephrology unit [CKD population used for denominator] | Pre-renal AKI or mild ATN not requiring follow up | PN services (all) | <17 | eGFR <90 ml/min/1.73m^2^ | 315 |
| Epidemiology and clinicopathologic outcome of pediatric chronic kidney disease in Nigeria, a single centre study | Olowu^44^ | 2013 | Nigeria | Retrospective single-centre | Review of medical records | To determine CKD incidence, prevalence, aetiologies, stages, clinicopathologic manifestations and outcome in Nigerian Children managed at centre | 10.0 | All new patients with laboratory or radiological evidence of CKD | Patients with insufficient lab data or <3 months follow-up | PN services (all) | <17 | Clinical evidence of CKD for ≥3 months; all stages 1-5 reported | 154 |
| Chronic Kidney Disease in children as seen in a tertiary hospital in Enugu, South-East Nigeria | Odetunde^23^ | 2014 | Nigeria | Retrospective single-centre | Review of medical records | Provide epidemiological data to inform health policies for children with CKD | 5.0 | Hospitalised children with CKD diagnosis | NR | Hospital admissions | <17 | Clinical evidence of CKD or eGFR <60 ml/min/1.73m^2^ for ≥3 months | 98 |
| Chronic kidney disease (CKD) in children in a Senegalese paediatric hospital | Keita^48^ | 2014 | Senegal | Retrospective single-centre | Review of medical records | To evaluate the epidemiological, clinical aspects and problems of managing CKD. | 9.0 | Children with GFR <60 presenting to the paediatric unit | AKI and patients with GFR >60 | PN services (all) | <16 | eGFR <60 ml/min/1.73m^2^ for >3 months | 53 |
| Chronic kidney disease among children in Guatemala | Cerón^47^ | 2014 | Guatemala | Prospective single-centre | Registry data | Describe the distribution of CKD in Guatemala, causes and age distribution, provide estimates of incidence and prevalence rates and estimate time to progress to ESRD | 9.0 | Patients presenting for care at FUNDANIER | NR | PN services (all) | <20 | eGFR <90 ml/min/1.73m^2^ | 432 |
| Predictors of renal replacement therapy and mortality in children with chronic kidney disease | Kari^49^ | 2015 | Saudi Arabia | Retrospective single-centre | Review of medical records | To examine risk factors for RRT and mortality among children with CKD | 9.0 | All children diagnosed with CKD in study period | AKI | PN services (all) | <15 | CKD stages 1-5 | 1000 |
| Causes of chronic kidney disease in Egyptian children | Safouh^50^ | 2015 | Egypt | Retrospective multi-centre | Review of medical records | To determine the etiology of CKD in Egyptian children and compare the results to other paediatric populations | 2.0 | New cases of CKD | AKI | PN services (all) | <20 | CKD stages 1-5 | 1018 |
| Non-medical risk factors as avoidable determinants of excess mortality in children with CKD. A prospective cohort study in Nicaragua, a model low income country | Montini^53^ | 2016 | Nicaragua | Retrospective single-centre | Registry data | To prospectively describe a cohort of children with CKD from Nicaragua. | 9.0 | All children diagnosed with CKD and referred to the tertiary hospital | SES data not available (n=52) | PN services (all) | <19 | CKD stages 1-5 | 257 |
| Pattern of renal diseases in children: A developing country experience | Yadav^54^ | 2016 | Nepal | Prospective single-centre | Review of medical records | To find out the pattern, clinical spectrum and outcome of patients of renal diseases at a tertiary care teaching hospital in Eastern region of Nepal | 1.0 | All cases of renal disease admitted to ward or reporting to renal clinic. [Denominator of CKD/CRF used] | <1 month old and non-renal disease | PN services (all) | <16 | NR | 3 |
| Renal diseases in children attending pediatric nephrology centres of Dhaka city | Qader^51^ | 2016 | Bangladesh | Retrospective multi-centre | Review of medical records | To observe the pattern of renal diseases in children of pediatric nephrology centres of Dhaka city | 1.0 | All cases of renal disease admitted to four hospitals across Dhaka city [Denominator of CKD/CRF used] | NR | PN services (all) | <19 | Clinical evidence of CKD or eGFR<90 ml/min/1.73m^2^ for >3 months | 70 |
| Chronic renal failure in Jamaican children: 2007-2012 | Miller^52^ | 2016 | Jamaica | Retrospective multi-centre | Review of medical records and questionnaire to local hospitals for non-referred cases of CRF. | To document all new cases of CRF in Jamaican children <12 years between 2007-2012, providing current data about the incidence and causes of CRF locally and dialysis availability. | 6.0 | All children newly diagnosed with chronic renal failure | NR | Country-wide survey/PN services (all) | <12 | eGFR <60 ml/min/1.73m^2^ for >3 months or less if clinical evidence of CKD | 27 |
| Epidemiology and outcomes of children with renal failure in the pediatric ward of a tertiary hospital in Cameroon | Halle^55^ | 2017 | Cameroon | Retrospective single-centre | Review of medical records | To describe the epidemiology and outcomes of children with renal failure in Cameroon | 10.0 | All children aged with renal failure admitted to pediatric ward | NR | Hospital admissions | <18 | eGFR <60 ml/min/1.73m^2^ for >3 months | 16 |
| Decreased rate of CKD stage V at admission among children: a single centre experience from the western part of Turkey | Alparslan^57^ | 2017 | Turkey | Retrospective single-centre | Review of medical records | To determine the changing characteristics of stages 3-5 CKD at admission to centre | 17.0 | Children admitted to centre with CKD | Inadequate records (n=10) | PN services (all) | <19 | eGFR <60 ml/min/1.73m^2^ for >3 months | 242 |
| Clinical and etiological profile of renal failure in children | Sandanala^56^ | 2018 | India | Prospective single-centre | Review of medical records | To study the clinical and aetiological profile of renal failure outcomes of patients with renal failure | 1.5 | Children presenting to paediatric department with chronic renal failure | <3 months, >18 years, trauma or post-operative cases | Pediatric department | <19 | eGFR <50 ml/min/1.73m^2^ for >3 months | 10 |
| Paediatric end-stage renal disease in a tertiary hospital in SW Nigeria | Asinobi^63^ | 2014 | Nigeria | Retrospective single-centre cohort | Review of medical records | Determine the incidence, aetiology and management/outcomes of paediatric ESRD in a Nigerian tertiary hospital | 8.0 | Incident patients managed for end-stage renal disease | NR | PN services (all) | <18 | Requiring KRT or death from kidney failure (with clinical evidence of CKD) | 53 |
| Does late referral to a nephrologist constitute a problem in children starting RRT in Poland? | Jander^60^ | 2006 | Poland | Retrospective multi-centre | Review of medical records | Assess the clinical and biochemical status of children starting RRT with respect to their timing of referral | 2.0 | Incident dialysis patients | Failure to recover after AKI | PN services (all) | <19 | Starting KRT | 180 |
| Causes and outcome of late referral of children who develop ESRD | Kennedy^58^ | 2012 | Australia | Retrospective single-centre | Review of medical records | Assess renal function of children who went on to develop ESKD, identify risk factors for late referral and to determine whether late referral influences outcomes; secondary aim to explore alternative definitions of late referral | 12.2 | Incident children with ESKD starting RRT | Patients <90 days of age | PN services (all) | <18 | Starting KRT | 47 |
| Early requirement for RRT in children at presentation in UK | Pruthi^11^ | 2016 | UK | Retrospective multi-centre | Registry data | Describe rates and factors influencing late referral in RRT population and investigate association with access to transplant and patient survival | 17.0 | Incident children with ESKD starting RRT | <3 months age, missing date of first PN review (n=138) | PN services (all) | <16 | Starting KRT | 1603 |
| Late referral impairs access to pre-emptive kidney transplantation in children | Boehm^59^ | 2010 | Austria | Retrospective single-centre | Review of medical records | To explore hypothesis that delayed referral impairs access to pre-emptive transplantation | 28.0 | Incident children with ESKD starting RRT | Acute presentation who were immediately started on dialysis and infants <8kg (number not given) | PN services (all) | <18 | Starting KRT | 111 |
| USRDS 2008 Annual Report | USRDS^64^ | 2008 | USA | Multi-centre | Registry data |  | 1.0 | Incident children with ESKD starting RRT | NR | PN services (all) | <21 | Starting KRT | 1231 |
| Determinants of eGFR at start of renal replacement therapy in paediatric patients | van Stralen^61^ | 2010 | Europe | Retrospective | Registry data | Aim to determine eGFR at start of RRT across Europe, determine how many patients were treated according to guidelines and investigate factors that determine GFR at RRT start and the rate of decline in the period prior to start of RRT | 6.0 | Incident children with ESKD starting RRT: data available from UK, Slovakia, Macedonia, Portugal, Lithuania, Czech Republic | Patients with missing eGFR data at first visit | PN services (all) | <19 | Starting KRT | 327 |
| Renal disorders in children: a Nigerian study | Eke^62^ | 1994 | Nigeria | Prospective single-centre | Proforma collected at admission | To investigate the prevalence and significance of renal disorders in a third world country with no facilities for paediatric RRT | 5.0 | Children presenting to outpatients or emergency ward with symptoms suggestive of renal disorders | NR | PN services (all) | <16 | NR | 15 |

Abbreviations: AKI, Acute Kidney Injury; ATN, acute tubular necrosis; CrCl, creatinine clearance; CRF, chronic renal failure; CKD, chronic kidney disease [*NB: Terminology used reflects that of study. CRF and CKD terms used interchangeably*]; ESRF, End-Stage Renal Failure; ESRD, End-Stage Renal Disease [*NB: Terminology used reflects that of study. ESRF and ESRD terms used interchangeably*]; eGFR, estimated Glomerular Filtration Rate; NAPRTCS, North American Pediatric Trials and Collaborative Studies; NR, not recorded; PN, Paediatric Nephrology; RRT, Renal Replacement Therapy *[NB: Term reflects terminology of study and is used interchangeably with Kidney Replacement Therapy]*; SD, standard deviation; SES, socio-economic status; USRDS, United States Renal Data System. *Numbers of excluded patients are provided where available.
