# Supplementary material for: The incidence of and risk factors for late presentation of childhood chronic kidney disease: A systematic review and meta-analysis
Source: PLoS One. 2020 Dec 31;15(12):e0244709. doi: 10.1371/journal.pone.0244709 (PMC7774987; doi:10.1371/journal.pone.0244709)
Supplement: Attachment — Submitted filename: Reviewers comments for PLOS ONE (3).docx [file pone.0244709.s007.docx]

Reviewer’s comments on the manuscript submitted to PLOS ONE

Manuscript number: PONE-D-20-29742

Title: The incidence of and risk factors for late presentation of childhood chronic kidney disease: a systematic review and meta-analysis

General comments

The authors of this manuscript conducted a systematic review and meta-analysis to determine the incidence of late presentation of chronic kidney disease (CKD) in children and its associated risk factors. After an in-depth and a well-researched review with good statistical analysis, they found a median incidence of 2.1 per million age-related population (pmrap), with non-congenital kidney diseases and older age as related risk factors. Undoubtedly, late stage of CKD, especially end-stage renal failure (ESRF) or end-stage kidney disease (ESKD), contributes to the global health burden in children. This underscores the need for a comprehensive data which reports the extent of this non-communicable disease. However, there are few specific concerns that should be addressed by the authors to improve the manuscript.

Specific comments

1. Abstract- Under Background, I think the term ‘kidney failure’ in line 25 should read ‘end-stage renal failure’ or ‘end-stage kidney disease’ since CKD progresses from stages 1 to 4 CKD and ends up in stage 5 CKD or end-stage renal failure. This observation also applies for ‘kidney failure’ mentioned in line 35 (under Results), although you stated in Table 2 that both terms are synonymous..
2. Introduction- Similarly, I suggest you replace the term ‘kidney failure’ in line 57 (and elsewhere in the manuscript) with ‘end-stage renal failure’ or ‘end-stage kidney disease’ and ‘kidney replacement therapy’ (KRT) with ‘renal replacement therapy’ (RRT). In lines 69 and 74 (and elsewhere in the manuscript), using the abbreviation ‘LP’ for late presentation is unnecessary and could be deleted more so when the same abbreviation also refers to one of the authors! (Line 115).
3. Materials and Methods- Table 1 on the systematic review criteria appears redundant and could be deleted. Presenting the information in prose form may suffice.
4. Discussion- In line 354, do you mean ‘guidance’ or ‘guideline’? Clarify and correct.
